# The Hawaiian Algal Database: a laboratory LIMS and online resource for biodiversity data

**DOI:** 10.1186/1471-2229-9-117

**Published:** 2009-09-04

**Authors:** Norman Wang, Alison R Sherwood, Akira Kurihara, Kimberly Y Conklin, Thomas Sauvage, Gernot G Presting

**Affiliations:** 1 Department of Molecular Biosciences and Bioengineering, 1955 East-West Road, University of Hawaii, Manoa, Honolulu, Hawaii, 96822, USA; 2 Department of Botany, 3190 Maile Way, University of Hawaii at Manoa, Honolulu, Hawaii, 96822, USA

## Abstract

**Background:**

Organization and presentation of biodiversity data is greatly facilitated by databases that are specially designed to allow easy data entry and organized data display. Such databases also have the capacity to serve as Laboratory Information Management Systems (LIMS). The Hawaiian Algal Database was designed to showcase specimens collected from the Hawaiian Archipelago, enabling users around the world to compare their specimens with our photographs and DNA sequence data, and to provide lab personnel with an organizational tool for storing various biodiversity data types.

**Description:**

We describe the Hawaiian Algal Database, a comprehensive and searchable database containing photographs and micrographs, geo-referenced collecting information, taxonomic checklists and standardized DNA sequence data. All data for individual samples are linked through unique accession numbers. Users can search online for sample information by accession number, numerous levels of taxonomy, or collection site. At the present time the database contains data representing over 2,000 samples of marine, freshwater and terrestrial algae from the Hawaiian Archipelago. These samples are primarily red algae, although other taxa are being added.

**Conclusion:**

The Hawaiian Algal Database is a digital repository for Hawaiian algal samples and acts as a LIMS for the laboratory. Users can make use of the online search tool to view and download specimen photographs and micrographs, DNA sequences and relevant habitat data, including georeferenced collecting locations. It is publicly available at .

## Background

Biodiversity inventories of defined geographical areas are critical resources for understanding the floristic or faunal composition of a region and provide a set of baseline data against which later collections can be compared. Most biological specimens resulting from such inventories are stored in private collections, museums, or herbaria that are not easily accessible to the general public. Collections housed in geographically isolated regions, such as the Hawaiian Islands, can be difficult to examine by scientists in other parts of the world. The Internet provides an excellent mechanism for disseminating specimen data.

As part of a biodiversity survey of the Hawaiian red algae, or Rhodophyta, we have been collecting and characterizing a large number of samples from the entire archipelago over the past three years. Specimens are collected from marine, freshwater and terrestrial habitats in Hawaii and collecting locations are georeferenced using GPS. Most of the specimens are stored as dried herbarium sheets or formalin vouchers in the Sherwood laboratory, and will ultimately be deposited in the Bernice Pauahi Bishop Museum (BISH). Specimens are photographed (both macro- and microscopically) to illustrate key identification characteristics, and DNA is extracted from most newly collected and select archived samples of the *Herbarium Pacificum *collection of BISH to generate DNA sequence data for organellar and nuclear markers. These data are critical for evaluating the molecular similarity of the Hawaiian red algal collections to those in other locations and for ascertaining the number of distinct genetic lineages of each species in the Hawaiian Islands.

To make these data available, we have built a custom-designed internet-accessible database, the Hawaiian Algal Database (HADB). HADB serves as both our internal laboratory information management system (LIMS) and a public portal for accessing the biodiversity survey data.

## Construction and content

The Hawaiian Algal Database was built using MySQL 5 and PHP 5 to store and display the various types of data collected during our biodiversity survey of red algae of the Hawaiian Islands. The web interface is programmed in PHP, using Smarty Template Engine to separate content from presentation. It meets the W3C XHTML 1.0 Strict specification drafted by the World Wide Web consortium. As a result, the web page will display correctly in any browser that conforms to the standard. The web interface also makes use of cascading style sheets (CSS) and is designed to be usable in browsers that do not support graphics. The interface includes a normal user interface for browsing only, and an administrator functions overlay for logged-in users with adequate privileges. The data input link and pages displayed in the administrator mode are double checked for sufficient privilege when executing the functions for any data alteration operations. Various table columns have additional indices constructed to optimize complex join query search speed. The database schema has been normalized into third normal form to avoid storage of data with logical inconsistencies. The image data are stored within the database as binary large objects (BLOB) for ease of resizing, watermarking and backup. Multiple versions of photos are not stored on disk; instead, they are generated on-the-fly upon request. For future scalability, caching can be implemented to speed up the image loading process. There is currently no noticeable lag due to on-the-fly image resizing and watermarking.

**Figure 1:**
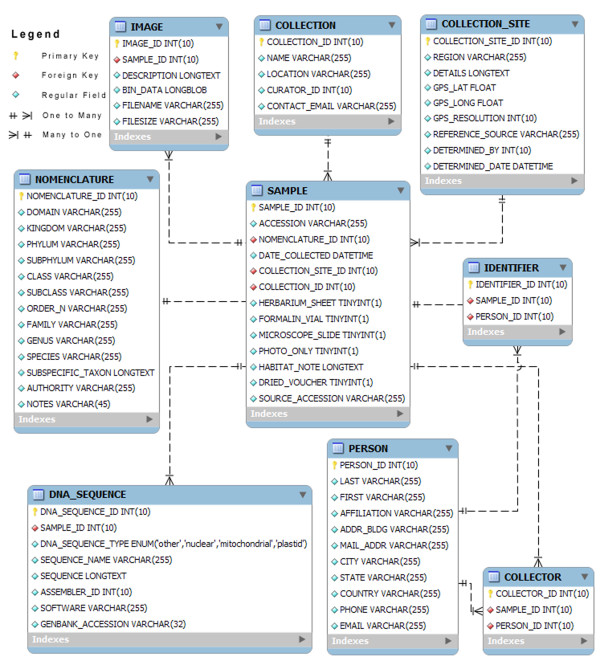
**The database schema**. Database schema: organization of the Hawaiian Algal Database and relationships between the nine tables.

The database uses Linux CentOS 4 and LAMP backend (Linux, Apache, MySQL, and PHP). The MySQL database is segmented into nine tables: COLLECTION, COLLECTION_SITE, COLLECTOR, DNA_SEQUENCE, IDENTIFIER, IMAGE, NOMENCLATURE, PERSON, and SAMPLE (Fig. 1). Web traffic is tracked at three levels: remote scripts, local scripts and server logs. General user traffic is monitored via cross-site scripts from Google Analytics. Local scripts monitor user-specific traffic via PHP sessions and apache server logs provide overall web server access statistics.

The HADB server is housed in a cooled, dehumidified server room at 20°C. The server utilizes an Intel Core 2 Duo 6600 processor, and is fitted with 6 GB of DDR2 RAM. The rack mount server case contains eight hot swappable hard disk bays and is housed in a water resistant server rack. Surge protection and uninterruptible power supply are provided. A second power supply is available in case of failure.

At any given time, at least three copies of the database are in existence to safeguard against catastrophic hardware failure or hacking. The data is both mirrored in real-time using software RAID 1 and backed up incrementally onto the hardware RAID 6 disk array. The hardware RAID 6 includes two spare drives and is operational with up to two concurrent disk failures. The capacity of the raid array is (N-2) * size of the smallest drive in the array. The incremental backup is performed by custom rsync shell script where unchanged files are copied as hard links to save on storage space, while only the delta of changed files is copied to minimize the time required to transfer the files. The scripts are automatically launched by a set of cron scripts every six hours up to a day, every day up to a week, every week up to four weeks, and every month up to 12 months.

## Utility and Discussion

Our goal was to build an open-access reference database that provides access to algal diversity information for the Hawaiian Islands. The database was designed to accommodate the types of data that are routinely obtained through biodiversity surveys and is unique in its accession-based design. As of this writing, HADB contains data for 2,211 accessions, of which 1,536 are represented by at least one photograph or micrograph, with a total of 2,917 images. Algae were collected from 333 distinct collection sites by 148 collectors and identified by 60 researchers. HADB presently contains 2,366 DNA sequences: 831 plastidial, 635 mitochondrial, and 900 nuclear sequences. The samples in the database represent 408 unique species, mostly red algae, and this number will continue to increase over the next several years. Specimen data are being shared with the Global Biodiversity Information Facility (GBIF).

Comparison of algal species collected in different geographic regions, at various times of the year, or by different collectors can be frustrating because key morphological features may vary with environmental conditions or be altogether absent (e.g. if the sexual stage is absent). DNA marker technology in the form of short DNA sequences from standardized regions of the genome can provide valuable information for linking samples across time and space [1]. We employ several DNA barcode-like markers in our biodiversity survey of Hawaiian red algae and are making these sequences and associated specimen data available through HADB as a reference database. These data will help researchers confirm taxonomic identification of algae, including those that are difficult to distinguish morphologically.

### Database use

Users are presented with a simple search interface. Detailed instructions appear upon placing the mouse cursor over the web interface. Users can search for samples in the database using several criteria including nomenclature fields ("Genus Species", Species, Genus, Family, Order, Subclass, Class and Subphylum), or locality searches ("Region", or island, and "Details", or detailed location). Individual samples can be found by searching with the Accession option from the drop down menu. Multiple accessions can be queried by entering accession numbers separated by a single space character.

The search result presents the user with 10 columns of information: Accession, Organism, Region, Details, Date, Collection and four DNA sequence columns. These 10 columns contain basic sample information for users to further narrow down samples they would like to view in more detail. The last four columns list the number of sequences for the sample per DNA sequence type: "N" for nuclear loci (e.g. partial 28S rRNA gene, or LSU [2]), "M" for mitochondrial loci (e.g. 5' cytochrome oxidase subunit I gene, or COI [3]), "P" for plastidial loci (e.g. partial 23S rRNA gene, or UPA;[4,5]), and "O" for other (e.g. any other locus).

**Figure 2:**
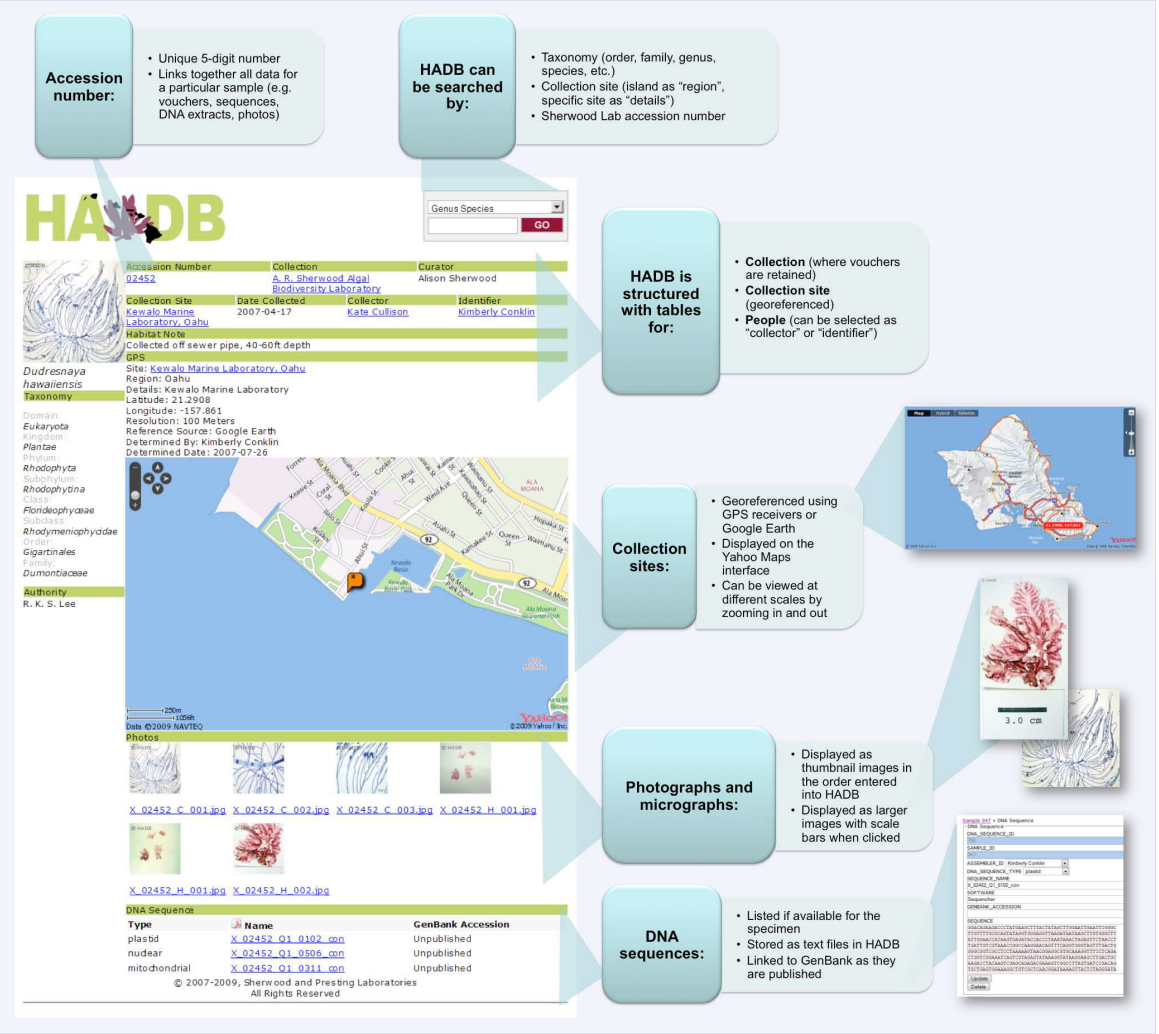
**Example database accession page**. Screenshot of an accession page illustrating the types of data stored for individual accessions and searchable fields.

The accession page displays a full range of data including taxonomy, collection, curator, date collected, collector, identifier, and collection site details (Fig. 2). The accession pages also include thumbnails of photographs or micrographs (which can be viewed at higher resolution by selecting them) and names of DNA sequences and GenBank accessions, if available. DNA sequences are accessible by clicking on the file name.

Precise GPS coordinates were determined for most newly visited collection sites using a Garmin GPSmap 76S receiver, or estimated based on high-resolution Google maps for accessions from archived collections and other samples that lacked GPS coordinates. These GPS coordinates are used to display a live-zoomable map of the collection site within the accession page via the Yahoo! Maps API.

## Conclusion

It is our hope that ecologists and taxonomists from the Pacific region and around the world will find this database useful for collaborative and comparative work on large biogeographic scales. The DNA sequences, in particular, should enable scientists to compare samples collected anywhere in the world to those found in the Hawaiian Islands. Detailed images and habitat data provide additional information to confirm DNA-based identifications and enable comparisons of habitat characteristics from different regions of the world. While HADB currently contains primarily members of the red algae, we expect to soon increase the number of accessions of other macroalgae (green and brown) and microalgae collected in the Hawaiian Islands. Use of HADB by laboratory members has demonstrated its value as a LIMS for organizing the diverse data types that are generated through organism-based biodiversity surveys.

## Availability and requirements

The Hawaiian Algal Database is publicly available at . The template MySQL database and PHP scripts are available upon request from the authors.

## Authors' contributions

NW modified a preliminary MySQL schema designed by Jeffrey Lai, and implemented the MySQL database, PHP scripts, interface design, and compiled database statistics. AK, KC, TS and ARS collected specimen data and entered them into HADB, data integrity checks were performed by ARS. ARS and GGP served as project advisors. NW, ARS and GGP wrote the manuscript. All authors checked the accuracy of the database and web interface, read and approved the final manuscript.
